# Using Reverse Osmosis Membrane at High Temperature for Water Recovery and Regeneration from Thermo-Responsive Ionic Liquid-Based Draw Solution for Efficient Forward Osmosis

**DOI:** 10.3390/membranes11080588

**Published:** 2021-07-31

**Authors:** Eiji Kamio, Hiroki Kurisu, Tomoki Takahashi, Atsushi Matsuoka, Tomohisa Yoshioka, Keizo Nakagawa, Hideto Matsuyama

**Affiliations:** 1 Research Center for Membrane and Film Technology, Department of Chemical Science & Engineering, Kobe University, 1-1 Rokkodai, Nada, Kobe 657-8501, Japan; e-kamio@people.kobe-u.ac.jp (E.K.); 170t420t@stu.kobe-u.ac.jp (H.K.); t.takahashi@crystal.kobe-u.ac.jp (T.T.); amatsuoka@people.kobe-u.ac.jp (A.M.); 2 Research Center for Membrane and Film Technology, Graduate School of Science, Technology, and Innovation, Kobe University, 1-1 Rokkodai, Nada, Kobe 657-8501, Japan; k.nakagawa@port.kobe-u.ac.jp (K.N.); tom@opal.kobe-u.ac.jp (T.Y.)

**Keywords:** forward osmosis membrane process, thermo-responsive draw solution, ionic liquid, lower critical solution temperature, osmotic pressure

## Abstract

Forward osmosis (FO) membrane process is expected to realize energy-saving seawater desalination. To this end, energy-saving water recovery from a draw solution (DS) and effective DS regeneration are essential. Recently, thermo-responsive DSs have been developed to realize energy-saving water recovery and DS regeneration. We previously reported that high-temperature reverse osmosis (RO) treatment was effective in recovering water from a thermo-responsive ionic liquid (IL)-based DS. In this study, to confirm the advantages of the high-temperature RO operation, thermo-sensitive IL-based DS was treated by an RO membrane at temperatures higher than the lower critical solution temperature (LCST) of the DS. Tetrabutylammonium 2,4,6-trimethylbenznenesulfonate ([N_4444_][TMBS]) with an LCST of 58 °C was used as the DS. The high-temperature RO treatment was conducted at 60 °C above the LCST using the [N_4444_][TMBS]-based DS-lean phase after phase separation. Because the [N_4444_][TMBS]-based DS has a significantly temperature-dependent osmotic pressure, the DS-lean phase can be concentrated to an osmotic pressure higher than that of seawater at room temperature (20 °C). In addition, water can be effectively recovered from the DS-lean phase until the DS concentration increased to 40 wt%, and the final DS concentration reached 70 wt%. From the results, the advantages of RO treatment of the thermo-responsive DS at temperatures higher than the LCST were confirmed.

## 1. Introduction

Recently, water treatment using forward osmosis (FO) membranes has attracted significant attention. Conventional membrane separation processes, such as reverse osmosis (RO), nanofiltration (NF), and ultrafiltration (UF), are pressure-driven separation methods that permeate water using mechanical pressure as the driving force for water permeation. The FO membrane process is osmotic pressure-driven [1]; herein, a semipermeable FO membrane is arranged between the draw solution (DS) with higher osmotic pressure and feed solution (FS) with lower osmotic pressure than that of DS; subsequently, water permeates through the FO membrane from FS to DS by the osmotic pressure difference generated between the two solutions. Therefore, mechanical pressure is not required to draw water from the FS through the FO membrane, and the pump power of the FO process is smaller than that of the pressure-driven membrane separation process. In addition, for the FO process, membrane fouling, which is one of the largest problems in the membrane separation method, has less influence than the pressure-driven membrane processes; therefore, the FO membrane is expected to be used for an extended period of time [2]. Owing to these advantages, the FO process is expected to be a key technology for realizing energy-saving water treatment processes for various applications such as seawater desalination [3,4,5], wastewater treatment [6,7,8], and food concentration [9,10].

However, to produce pure water by the FO process, it is necessary to separate water from the diluted DS after water permeation from FS through the FO membrane. The energy required to recover water from the diluted DS accounts for most of the energy consumed in the FO process. In other words, separating water from diluted DS with less energy is a challenge that needs to be overcome for realizing an efficient energy-saving water production process using an FO membrane; therefore, it is important to use an appropriate DS for the water production process [11].

To date, various DSs have been proposed [12], including inorganic salts such as NaCl, MgCl_2_, MgSO_4_, and KCl [13,14,15,16], natural sugars [3,17], fertilizers [18,19,20], polymer electrolytes [21,22], zwitterion [23], hydrogels [24], and carbon quantum dots [25]. If the FO process is applied to pure water production, recovery of water from diluted DS is necessary. The energy required to recover water from diluted DS accounts for most of the energy required for the entire process of water production by FO. For FO processes that use MgCl_2_, MgSO_4_, and natural sugars as DS, FO-RO, and FO-NF, hybrid processes have been proposed [13,14,15,16]. The hybrid process uses RO or NF to recover water from the diluted DS. It has been reported that the energy cost required for the hybrid process is lower than the direct desalination of seawater by RO. On the other hand, the use of stimulus-responsive DS has also been proposed as a useful option for low-energy water production processes.

**Figure 1:**
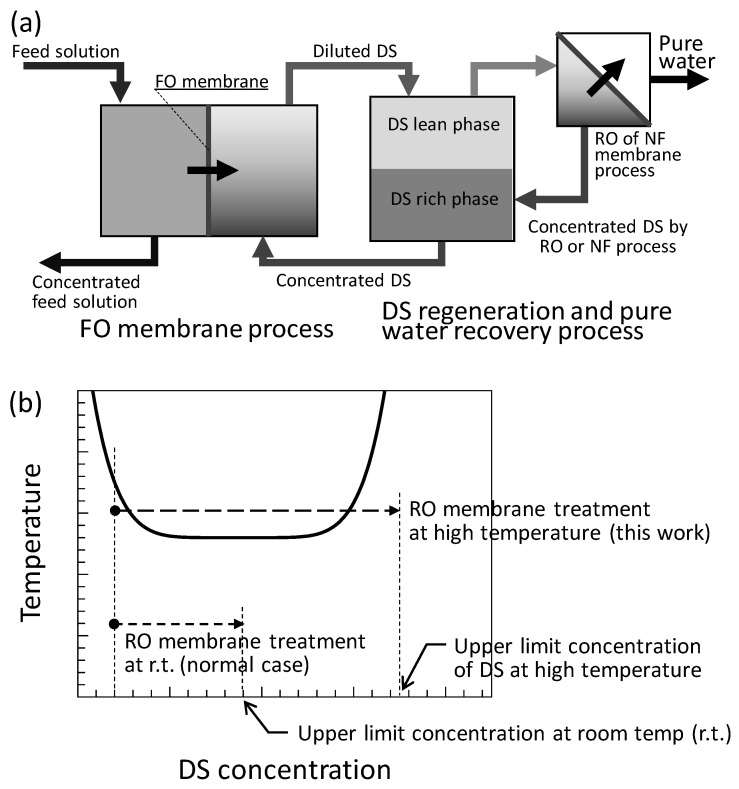
(**a**) Schematic of the FO process involving DS regeneration and pure water recovery processes with an RO or NF membrane. (**b**) Schematic DS concentration change by RO membrane treatment.

Several kinds of stimuli-responsive DSs have recently been proposed and developed. To separate water from stimuli-responsive DSs, heat [26,27,28,29,30,31,32,33,34,35,36], magnetic force [37], pH [38], and CO_2_ [39,40,41] can be used as external stimuli. Among these stimuli-responsive DSs, thermo-responsive DS is the most widely studied. For example, an aqueous solution of ammonium bicarbonate is a thermo-responsive DS. Ammonium bicarbonate can be easily decomposed and removed from the DS by heating [35,36]. An aqueous solution of the thermo-responsive material can also be used as a thermo-responsive DS, which can be separated into DS-rich and DS-lean phases by controlling the temperature. To date, aqueous solutions of low-molecular-weight amine compounds [26], glycol ethers [27], low-molecular-weight polypropylene glycol [28], and thermo-responsive ionic liquids [29,30,33,34] have been reported as thermo-responsive DSs. When the thermo-responsive DS is used in the FO membrane process, the DS diluted by drawing water from the FS is heated to separate the DS-rich and -lean phases, and the water is recovered from the DS-lean phase by a low-pressure RO operation or NF membrane operation. The concentrated DS-lean phase is mixed with the DS-rich phase and reused for FO operation. A schematic of the FO membrane water treatment process using a thermo-responsive DS is shown in Figure 1a. For the thermo-responsive phase separation of the DS, low-grade waste heat from power plants and factories can be used. Therefore, the energy costs for water recovery and DS regeneration can be reduced. It is estimated that the FO process accompanying the RO treatment of the DS-lean phase can reduce the pump power by 84% compared to the direct RO treatment of seawater [31]. Furthermore, if the osmotic pressure of the DS-lean phase can be further reduced, the mechanical pressure required for the RO or NF treatment of the DS-lean phase can be further reduced.

Recently, we found that the osmotic pressure of thermo-responsive IL-DS is temperature-dependent and smaller at elevated temperatures [32]. In addition, we confirmed that a high-water flux could be realized by treating the DS-lean phase with an RO membrane at high temperatures and proposed the effectiveness of the high-temperature DS regeneration process.

In our previous work, the DS-lean phase was treated using an RO membrane at high temperature. However, these temperatures were lower than the lower critical solution temperature (LCST) of the DS. In this work, the RO membrane treatment of the DS-lean phase was investigated at temperatures higher than the LCST (Figure 1b), and the superiority of the high-temperature RO treatment of dilute DS was demonstrated. The high-temperature RO membrane treatment of the thermo-responsive DS, in which the osmotic pressure decreases at high temperatures, will not only increase the water flux but also raise the upper limit of the DS concentration by the RO treatment as shown in Figure 1b. In addition, in this study, we propose a further advantage of the high-temperature RO treatment of the DS-lean phase at temperatures higher than the LCST of the DS. Under the condition that the DS separates into DS-rich and -lean phases, the osmotic pressures of the two liquid phases were found to be equal. During the concentration of DS by RO treatment in the two-phase region of the liquid–liquid phase separation, the volume fractions of the DS-rich phase and the DS-lean phase vary, but their osmotic pressure does not change. Therefore, in the two-phase region, the driving force of water permeation, which is the pressure difference between the mechanical pressure applied to the FS and the osmotic pressure of the DS, for the RO membrane treatment does not increase but is constant. Therefore, at temperatures higher than the LCST, it is considered that a high-water flux for recovering water from the DS-lean phase can be maintained over a wide DS concentration range. In this study, we experimentally confirmed the advantages of the high-temperature RO membrane treatment of the DS-lean phase after liquid–liquid phase separation at temperatures higher than the LCST.

## 2. Experimental

### 2.1. Materials and Reagents

The heat-tolerant RO membrane (NTR-759HG) composed of polyamide active layer and polysulfone support was purchased from Nitto Denko Co. (Osaka, Japan). The maximum allowable temperature and pressure for continuous use are 60 °C and 49 bar, respectively. At 60 °C, the pure water flux was about 70 LMH, and the NaCl rejection for 0.11 mol/L NaCl aqueous solution was 94%, respectively. Tetrabutylammonium hydroxide (40% solution in water) ([N_4444_][OH]), sodium dimethylbenzenesulfonate monohydrate, sodium mesitylenesulfonate, and trifluoroacetic acid were purchased from Tokyo Chemical Industry Co., Ltd. (Tokyo, Japan). Tetrabutylphosphonium hydroxide (40% in water) was purchased from Sigma-Aldrich Co. (Tokyo, Japan). Pure water was generated using a Millipore Milli-Q system (Millipore, Burlington, MA, USA). All materials and reagents were used as received without further treatment or purification. Tetrabutylammonium 2,4,6-trimethylbenznenesulfonate ([N_4444_][TMBS]), tetrabutylphosphonium 2,4-dimethylbenzenesulfonate ([P_4444_][DMBS]), and tetrabutylammonium trifluoroacetate ([N_4444_][CF_3_COO]) were used to prepare thermo-responsive IL-DSs. They were synthesized in our laboratory using the same procedure reported in our previous study [30].

### 2.2. Evaluation of Osmotic Pressure of the LCST-Type Solution

The osmotic pressure of the aqueous solution of [N_4444_][TMBS] at 25 °C was measured using a water activity meter (AquaLab Seris4TDL, AINEX Co., Ltd., Japan). Aqueous solution samples (7 mL) were injected into the sample vial, maintained at 25 °C for more than 15 min, and the activities of water in each sample (*a_w_*) were measured. The measured *a_w_* was used to calculate the osmotic pressure, *π*, using the following equation:

(1) $$\pi =-\frac{RT\times lna_{w}}{V_{H2O}}=\frac{1-a_{w}}{M_{H_{2}O}/\rho_{H_{2}O}}RT$$

where *V*_H2O_ is the partial molar volume of water in the [N_4444_][TMBS] aqueous solution at 25 °C. *M*_H2O_ is the molecular weight of water, and *ρ*_H2O_ is the density of pure water. *R* is the gas constant, and *T* is the temperature. The osmotic pressures of [N_4444_][TMBS] aqueous solutions other than 25 °C, [P_4444_][DMBS] aqueous solutions, and [N_4444_][CF_3_COO] aqueous solutions were taken from Ref. [32].

### 2.3. Evaluation of RO Flux for the [N_4444_][TMBS] Aqueous Solution

[N_4444_][TMBS] was used as the DS solute for LCST phase separation materials. Referring to the phase diagrams, the concentration was designated as 8 wt%, corresponding to the composition of the lean phase at 70 °C. At temperatures of 25, 40, and 60 °C, the RO concentration was measured at a constant pressure of 15 bar, and the water permeation flux with respect to the change in solute concentration was measured. The [N_4444_][TMBS] concentrations in the DS during the RO operation were measured using an automated Karl Fischer titrator (Hybrid Karl Fischer Moisture Titrator MKH-700, Kyoto Electronics Manufacturing Co. Ltd., Kyoto, Japan).

**Figure 2:**
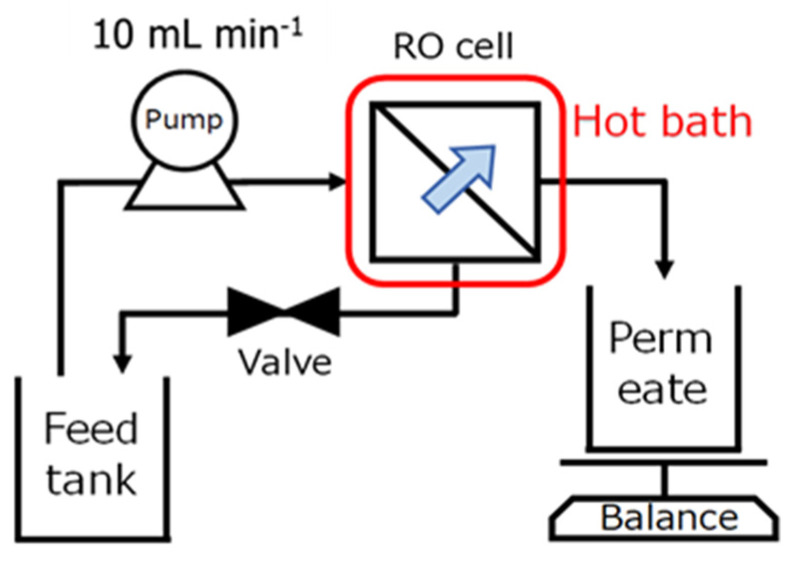
Schematic illustration for RO filtration apparatus.

The RO filtration setup is illustrated in Figure 2. A piece of heat-tolerant RO membrane was placed inside the thermostatted RO cell, which was immersed in a constant-temperature water bath for precise temperature control. The diaphragm pump was adjusted to provide the FS at a flow rate of 10 mL min^−1^, and the pressure was adjusted using a valve. The cell was constantly stirred with a magnetic stirrer to prevent concentration polarization at the membrane surface. The filtrate was automatically collected and weighed, and the stabilized mass data were used to calculate the water flux.

Because the polymeric RO membrane may deform at high temperatures, which could result in a potential impact on water permeance, we pre-treated the membranes by filtrating pure water at 60 °C and 15 bar for 12 h before each experiment. After the pretreatment, the water flux became almost constant at about 80% of the initial value. Thus, the effects of membrane deformation and consolidation can be eliminated.

## 3. Results and Discussion

### 3.1. Temperature Dependence of Osmotic Pressure of IL-Based DSs

**Figure 3:**
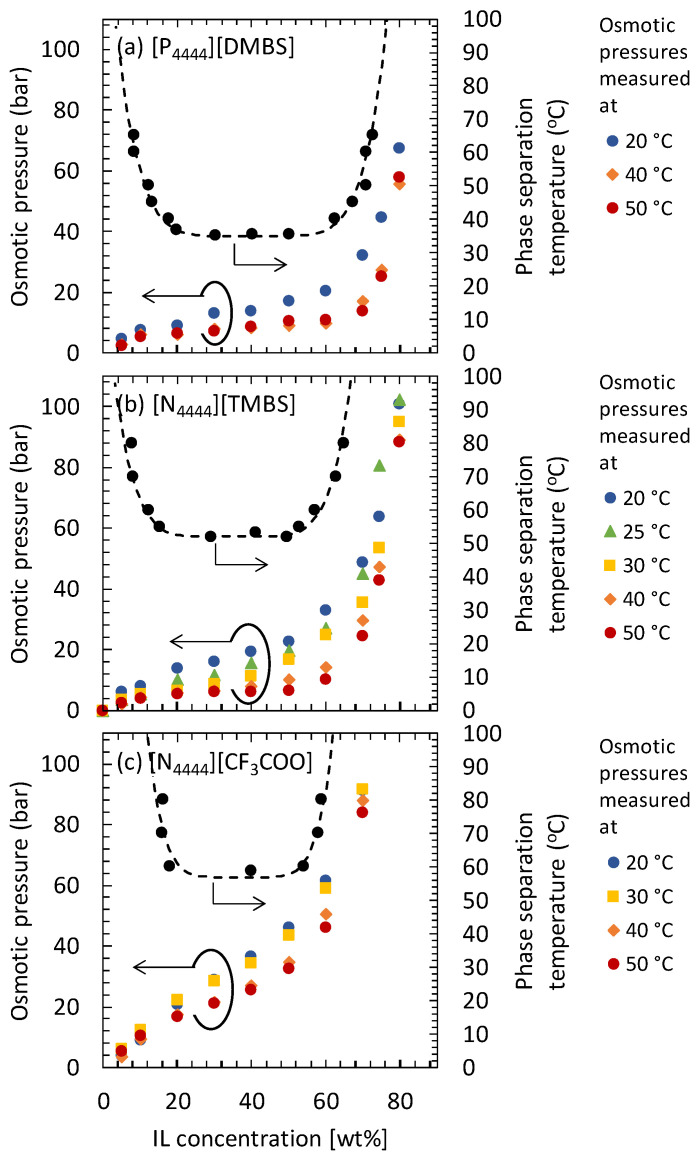
Phase diagram and temperature dependency of the osmotic pressure of thermo-responsible DS at different temperatures. (**a**) [P_4444_][DMBS], (**b**) [N_4444_][TMBS], and (**c**) [N_4444_][CF_3_COO]. The data other than the osmotic pressure of [N_4444_][TMBS] aqueous solution measured at 25 °C were those previously reported in Ref. [32]. Reproduced with permission from Ref. [32]. Elsevier, 2021.

Figure 3 shows the phase diagram of [P_4444_][DMBS], [N_4444_][TMBS], and [N_4444_][CF_3_COO] aqueous solutions and the osmotic pressures of each IL-DS at different temperatures. The osmotic pressure of each IL-DS decreased as the temperature increased. This is because the temperature-responsive IL forms aggregates at high temperatures and the concentration of free IL molecules in the DS decreases [32]. Focusing on the concentration dependence on osmotic pressure, the osmotic pressure at a temperature lower than the LCST increased monotonically as the IL concentration increased (for example, Figure 3c). In contrast, the osmotic pressure barely increases in the concentration range where the phase separation occurs (two-phase region). In the two-phase region, the DS separates into DS rich phase and DS lean phase. When the mole of IL in the DS-rich and -lean phases are respectively *n*_1_ and *n*_2_, and the volumes of the DS-lean and -rich phases are respectively *V*_1_ and *V*_2_, the IL concentration of the DS rich phase, *C*_1_, and that of the DS lean phase, *C*_2_, are *C*_1_ = *n*_1_/*V*_1_ and *C*_2_ = *n*_2_/*V*_2_, respectively. It should be noted that, in the two-phase region, the IL concentration shown in the x-axis of Figure 3 is an apparent IL concentration calculated from the total mole of IL in the total volume of the DS. That is, in the abovementioned case, the apparent IL concentration is calculated as (*n*_1_ + *n*_2_)/(*V*_1_ + *V*_2_). If the apparent IL concentration changed in the two-phase region, *n*_1_, *n*_2_, *V*_1_, and *V*_2_ are also changed, but the IL concentrations of the DS rich and lean phases, *C*_1_ and *C*_2_, are not changed. Thus, in the two-phase region, the osmotic pressures of the DS-rich and -lean phases are constant regardless of the apparent IL concentration. In addition, the DS-rich and -lean phases should have the same osmotic pressure. This is because the chemical potentials of H_2_O and IL in the DS-lean and -rich phases formed by phase separation are equal. In fact, in the case of [N_4444_][DMBS] aqueous solution, which has a two-phase region at 40 °C and 50 °C, the osmotic pressures in the two-phase regions at these temperatures were almost constant, as shown in Figure 3a. In other words, in the two-phase region at temperatures higher than the LCST, it is predicted that the driving force of water permeation from the DS-lean phase through RO or NF membranes (applied pressure–osmotic pressure) will be constant. Moreover, the osmotic pressure of the thermo-responsive IL aqueous solution decreases at high temperatures. Therefore, it is expected that the driving force of pure water permeation during high-temperature operation could be much larger than that during the low-temperature operation. In fact, in a previous study, we confirmed that the water flux from the DS was higher when the RO operation was performed at a high temperature [32]. Therefore, from the viewpoint of obtaining a high-water flux, it is suggested that high-temperature RO (or NF) membrane operation is effective for treating the DS-lean phase.

### 3.2. Effect of High-Temperature RO Treatment for DS Regeneration

The treatment of the DS-lean phase enables the regeneration of the DS-lean phase in addition to water recovery. For the regeneration of the DS-lean phase, the IL concentration should be increased as much as possible by RO (or NF) operation. Therefore, we evaluated the efficiency of high-temperature operation for DS-lean phase treatment by RO or NF membranes from the viewpoint of the IL concentration limit of the DS-lean phase.

**Figure 4:**
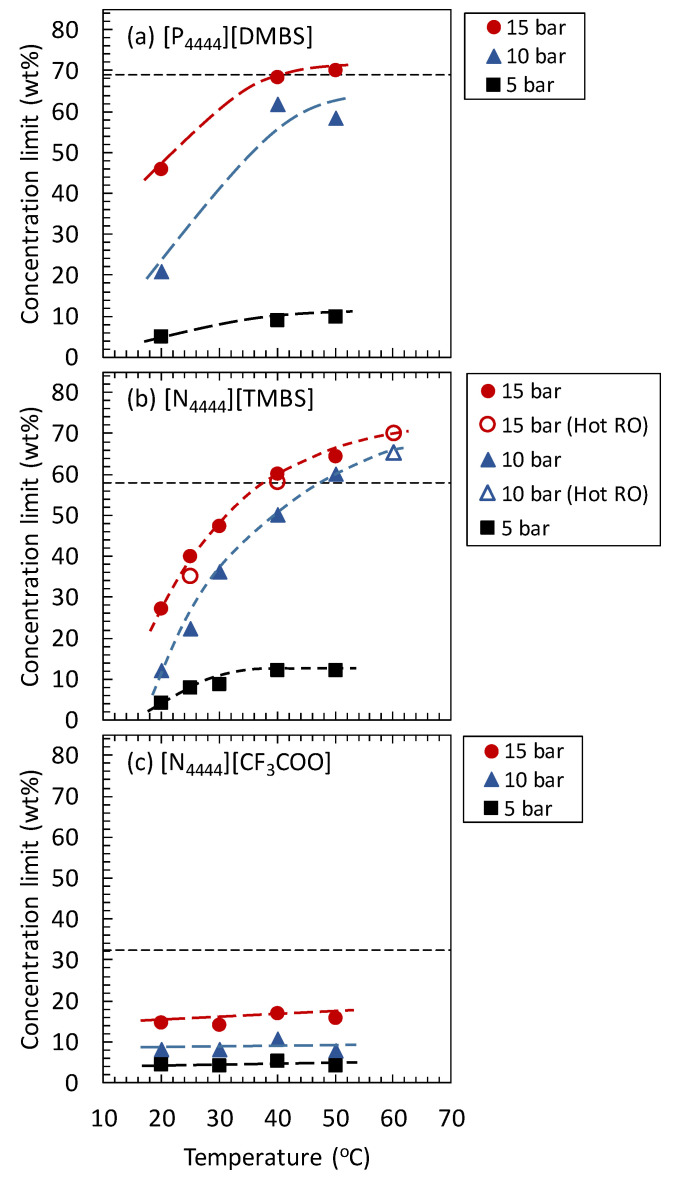
Effect of temperature on upper concentration limits of each IL-DS. (**a**) [P_4444_][DMBS], (**b**) [N_4444_][TMBS], and (**c**) [N_4444_][CF_3_COO]. The solid symbols correspond to results predicted from the relationship between osmotic pressure and IL concentration shown in Figure 3. The open symbols correspond to experimentally determined concentration limits of [N_4444_][TMBS] for RO under various pressure and temperature conditions.

For seawater desalination by FO, the osmotic pressure of the regenerated DS must be higher than the osmotic pressure of seawater (approximately 30 bar). In other words, the DS-lean phase should be concentrated such that its osmotic pressure is higher than that of seawater. For example, if seawater desalination is performed at 20 °C (room temperature), the minimum DS concentration required for the FO desalination operation can be estimated from the osmotic pressure at 20 °C, shown in Figure 3. The minimum IL concentrations required were approximately 69 wt%, 58 wt%, and 32 wt% for [P_4444_][DMBS], [N_4444_][TMBS], and [N_4444_][CF_3_COO], respectively (Figure S1). Furthermore, the upper concentration limit of the concentrated DS-lean phase treated by RO and NF membrane operation at a certain mechanical pressure and temperature can be determined from the osmotic pressure with respect to the operating temperature (Figure 3). For example, when concentrating an aqueous solution of [P_4444_][DMBS] at 50 °C at an operating pressure of 15 bar, the upper concentration limit of [P_4444_][DMBS] would be approximately 70 wt% (Figure S2). Similarly, the upper concentration limits of each IL-DS at various temperatures and operating pressures of 15, 10, and 5 bar were determined. These results are shown in Figure 4. This figure shows the effect of temperature on the upper concentration limits of each IL-DS concentrated using RO or NF membranes under various pressure conditions. In this figure, the required concentrations of ILs in each DS for seawater desalination at 20 °C are also shown as broken lines.

For all the IL-DSs examined in this study, the upper concentration limits increased with increasing mechanical pressure and temperature. This increase in the upper concentration limits is due to the decline in the osmotic pressure of thermo-responsive IL-DSs with an increase in temperature. Upon comparing the upper concentration limits of the IL-DSs at the same temperature, the order of the upper concentration limits was [P_4444_][DMBS] > [N_4444_][TMBS] > [N_4444_][CF_3_COO]. Moreover, as shown in Figure 3, the order of the osmotic pressure of the IL-DSs was [N_4444_][CF_3_COO] > [N_4444_][TMBS] > [P_4444_][DMBS] at the same IL concentration and temperature. That is, IL-DSs with a lower osmotic pressure have a higher upper concentration limit.

The efficiency of the seawater desalination process when using each IL-DS is discussed herein. As shown in Figure 4a, the concentration of the [P_4444_][DMBS]-based DS can be increased up to 70 wt% using RO or NF membranes under operating conditions of 50 °C and 15 bar. However, the osmotic pressure of the [P_4444_][DMBS]-based DS at 20 °C was extremely low (Figure 3a). Even at a concentration of 70 wt%, the osmotic pressure was almost the same as that of seawater at 20 °C (30 bar). Therefore, the [P_4444_][DMBS]-based DS is not suitable for seawater desalination.

The [N_4444_][CF_3_COO]-based DS has a high osmotic pressure in the temperature range of 20 °C to 50 °C (Figure 3c). Therefore, if the concentration is higher than 32 wt%, the [N_4444_][CF_3_COO]-based DS is capable of drawing water from seawater at 20 °C. However, because of the high hydrophilicity of [N_4444_][CF_3_COO], the [N_4444_][CF_3_COO]-based DS maintains a high osmotic pressure even at high temperatures. Thus, as shown in Figure 4c, the concentration of the [N_4444_][CF_3_COO]-based DS cannot exceed 15 wt% even under the operating conditions of 50 °C and 15 bar, and the osmotic pressure is lower than that of seawater. Thus, the [N_4444_][CF_3_COO]-based DS is not suitable for seawater desalination.

As shown in Figure 4b, by RO or NF at a high temperature, it is possible to increase the upper concentration limit of the [N_4444_][TMBS]-based DS above 58 wt%, which is the concentration at which the osmotic pressure is similar to that of seawater. For example, under the conditions of an operating pressure of at least 15 bar and a temperature of at least 40 °C, the upper concentration limit of [N_4444_][TMBS] can be increased to more than 58 wt%. Even at an operating pressure of 10 bar, [N_4444_][TMBS] can be concentrated to more than 58 wt% when the temperature is higher than 50 °C. This high upper concentration limit of the [N_4444_][TMBS]-based DS is due to the highly temperature-dependent osmotic pressure of the [N_4444_][TMBS] aqueous solution (Figure 3b). Therefore, from the viewpoint of the concentration limit and DS regeneration, the [N_4444_][TMBS]-based DS is promising, and the DS-lean phase can be preferably treated by RO (or NF) membranes at high temperatures.

### 3.3. Water Recovery from Diluted [N_4444_][TMBS] Aqueous Solution at High Temperature

**Figure 5:**
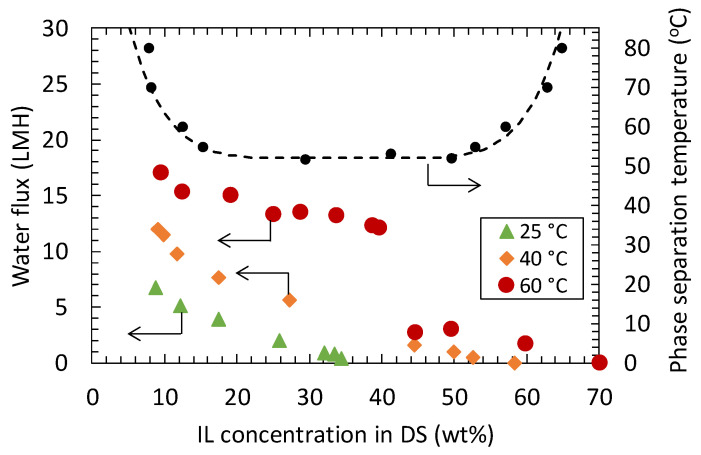
Relationship between the water flux and [N_4444_][TMBS] concentration in DS at different temperatures. The black plot with broken lines is the phase diagram of the [N_4444_][TMBS] aqueous solution.

From the temperature dependence of the osmotic pressure, it was found that the [N_4444_] [TMBS] aqueous solution is suitable for use in FO desalination. Further, it was suggested that the effective treatment of the [N_4444_][TMBS] lean phase by RO or NF membranes could be performed at high temperatures, in view of a high-water flux and high upper concentration limit for DS regeneration. To confirm the effectiveness of such a high-temperature RO process, RO treatment of the [N_4444_][TMBS] lean phase was conducted. In this investigation, an 8 wt% [N_4444_][TMBS] aqueous solution, which corresponds to the [N_4444_][TMBS] lean phase obtained by thermo-responsive phase separation at 70 °C, was used. Water permeation tests were conducted at an operating pressure of 15 bar at 25 °C, 40 °C, and 60 °C. The results are shown in Figure 5.

Figure 5 shows the relationship between the water flux and [N_4444_][TMBS] concentration in the DS, which was continuously changed by water permeation during the water permeation test. The phase diagram of [N_4444_][TMBS] is also shown in this figure. At 25, 40, 60 °C, the [N_4444_][TMBS] rejection were 98.8 ± 0.2%, 98.8 ± 0.3%, and 98.6 ± 0.5%, respectively. The final concentrations of [N_4444_][TMBS] capable of drawing water at 25, 40, and 60 °C were 35, 58, and 70 wt%, respectively. The [N_4444_][TMBS] recovery at 25, 40, and 60 °C were 96.0, 91.9, and 85.6%, respectively. The relationships between the final concentrations corresponding to the concentrations at the stop of water flux and the temperature of RO treatment are plotted as open symbols in Figure 4b. These data points were located on the master curves shown in Figure 4b, which were determined from the phase diagram of the [N_4444_][TMBS] aqueous solution. This indicated that water permeation in the RO operation was stopped when the osmotic pressure of the [N_4444_][TMBS] solution became equal to the applied mechanical pressure.

Furthermore, it is worth noting that the water flux at 60 °C was also substantially high. As shown in Figure 5, the initial water flux increases with increasing operation temperature. This is because of the low osmotic pressure of the DS and the low viscosity of the FS and DS at high temperatures [32]. In addition, the high-water flux at 60 °C was maintained until the [N_4444_][TMBS] concentration increased to 40 wt%. By contrast, at 25 °C and 40 °C, the water flux monotonically decreased with an increase in [N_4444_][TMBS] concentration. The monotonous decrease in water flux at 25 °C and 40 °C is a result of the monotonous increase in osmotic pressure with increasing DS concentration. At these temperatures, the [N_4444_][TMBS] aqueous solution did not phase-separate over the entire [N_4444_][TMBS] concentration range, and the osmotic pressure continuously increased with increasing [N_4444_][TMBS] concentration. Therefore, as the concentration of the DS increased, the driving force of water permeability decreased, and the water flux decreased monotonically. In addition, it is considered that the increase of the [N_4444_][TMBS] concentration would induce concentration polarization and membrane fouling to further decrease of the water flux.

The high water flux at 60 °C in the [N_4444_][TMBS] concentration range up to 40 wt% is due to the liquid–liquid phase separation caused by the increase in the [N_4444_][TMBS] concentration during the RO treatment. At 60 °C, the [N_4444_][TMBS] aqueous solution separated into a rich DS phase and a lean DS phase in the concentration range of 12–58 wt%. The [N_4444_][TMBS] concentrations of these rich and lean phases were constant at a constant temperature. In addition, because the rich and lean phases are at equilibrium, the chemical potentials of H_2_O and [N_4444_][TMBS] in these phases are equal. Therefore, the osmotic pressures of the rich and lean phases were the same and constant. In fact, Figure 3b shows that the osmotic pressure of the rich and lean DS phases at 60 °C is less than 5 bar, which is the osmotic pressure of a 12 wt% [N_4444_][TMBS] aqueous solution at 50 °C. Therefore, when the mechanical pressure for the RO operation was 15 bar, in the concentration range of 12–58 wt%, the pressure difference between FS and DS, which is the driving force for water permeation by the RO membrane, is maintained at more than 10 bar. Owing to the high and constant driving force, a high-water flux was maintained in the [N_4444_][TMBS] concentration range of 12 wt% to 40 wt%. In other words, from this result, it can be said that the concept of high-speed RO treatment under phase separation conditions was verified.

Another interesting phenomenon was observed during the RO at 60 °C, namely, a drastic decline in the water flux at the [N_4444_][TMBS] concentration of approximately 40 wt%. In addition, as shown in Figure 5, the decreased water flux remained almost constant until the [N_4444_][TMBS] concentration reached approximately 60 wt%. The drastic decline of the water flux and nearly constant water flux after the drastic decline cannot be explained by the continuous increase in the osmotic pressure along with the increase in the apparent [N_4444_][TMBS] concentration during the RO treatment. As shown in the phase diagram, at 60 °C, the [N_4444_][TMBS] aqueous solution separates into DS-rich and -lean phases. In the RO membrane cell, the two phases were vigorously agitated by a magnetic stirrer. Thus, the two phases would become an emulsion, and the continuous phase could cover the surface of the active layer of the RO membrane (Figure S3). When the apparent [N_4444_][TMBS] concentration was small, the continuous phase was the DS-lean phase (Figure S3a). However, the volume of the DS-rich phase gradually increased with the increase in the apparent [N_4444_][TMBS] concentration during the RO operation. When the volume of the DS-rich phase became larger than that of the DS-lean phase, it is considered that the transformation of the continuous phase and disperse phase could have occurred. Thus, the DS-rich phase could become the continuous phase and cover the active layer of the RO membrane (Figure 3b). We speculated that this phase transition would strongly affect the drastic water flux change during RO operation at 60 °C. As mentioned above, the concentrations of the rich and lean DS phases were 58 wt% and 12 wt% at 60 °C, respectively. Thus, when the apparent [N_4444_][TMBS] concentration was in the range of 12 wt% to approximately 35 wt% (half of the sum of 12 wt% and 58 wt%), the lean phase is the continuous phase, and the rich phase is the disperse phase, according to the phase separation theory [42,43]. In the apparent [N_4444_][TMBS] concentration range of approximately 35 wt% to 58 wt%, the opposite configuration occurs; that is, the rich phase becomes the continuous phase. The boundary concentration between the higher and lower water fluxes (40 wt% of the apparent [N_4444_][TMBS] concentration) observed in Figure 5 roughly corresponds to the concentration at which the phase configuration is transformed (approximately 35 wt%). From 12 wt% to 40 wt%, the lean phase mainly contacted the active layer of the RO membrane because the lean phase is the continuous phase in this apparent [N_4444_][TMBS] concentration range (Figure S3a). In contrast, the rich phase contacted the active layer of the RO membrane from 40 wt% to 58 wt% because the rich phase is the continuous phase in this apparent [N_4444_][TMBS] concentration range (Figure S3b). The sudden decrease in the water flux at 40 wt% was mainly due to the decrease in the diffusion rate of H_2_O in the DS near the surface of the RO membrane. The viscosity of the DS-lean phase was 0.92 mPa s, whereas that of the DS-rich phase was 5.26 mPa s. Thus, the diffusion coefficient of H_2_O in the lean phase is larger than that in the rich phase. Furthermore, when the lean phase is in contact with the membrane surface, the boundary layer thickness is smaller than when the rich phase is in contact with the membrane surface. In addition, from the phase diagram of the [N_4444_][TMBS] aqueous solution, it is estimated that the H_2_O concentrations of the lean and rich DS phases are 48.8 mol/L and 23.6 mol/L, respectively. Therefore, when the lean phase covers the membrane surface, the driving force for H_2_O diffusion in the boundary layer near the membrane surface would be large. Conversely, when the rich phase covers the membrane surface, the driving force would be small. Thus, it can be considered that the diffusion rate of H_2_O via the boundary layer of the membrane surface is higher when the lean phase is in contact with the membrane surface. Consequently, the water flux is drastically decreased, by approximately 40 wt%, upon changing the phase configuration.

According to the phase separation theory [42,43], at DS concentrations of 12 to 58 wt%, the volume ratio of the rich phase to the lean phase increases with increasing DS concentration. However, the concentrations of the lean and rich phases were maintained at 12 wt% and 58 wt%, respectively. Thus, the water fluxes were almost constant, at high levels, for DS concentrations of 12 wt% to 40 wt%. In addition, they were also constant at low levels for DS concentrations of 40 wt% to 58 wt%, as shown in Figure 5.

The change in the [N_4444_][TMBS] aqueous solution near the RO membrane surface from the lean phase to the rich phase is the reason for the rapid decrease in water flux. In this context, it is considered that the RO efficiency could be improved by surface modification of the RO membranes. That is, if an RO membrane with a highly hydrophilic surface can be developed, the DS-lean phase will preferentially cover the hydrophilic surface, even in the case of the isolated configuration of the DS-lean phase, and a high-water flux can be maintained over a wide concentration range. Improvement of the water flux by surface modification of RO membranes is a topic for future research.

## 4. Conclusions

The effect of high-temperature RO on water recovery and DS regeneration was investigated using thermo-responsive IL-DSs. Specifically, aqueous solutions of [P_4444_][DMBS], [N_4444_][TMBS], and [N_4444_][CF_3_COO] were used as the thermo-responsive IL-DSs. Because of the low-temperature dependence of the osmotic pressure of the [P_4444_][DMBS]-based and [N_4444_][CF_3_COO]-based DSs, the upper concentration limits of these IL-DSs were not sufficient to generate osmotic pressures higher than that of seawater at 20 °C. By contrast, the osmotic pressure of the [N_4444_][TMBS]-based DS was highly temperature-dependent. When RO was performed at 15 bar and 40 °C, the DS was concentrated, generating an osmotic pressure higher than that of seawater at 20 °C. In addition, because [N_4444_][TMBS]-based DS has low osmotic pressure and low viscosity at high temperatures, a substantially high-water flux was obtained at 60 °C. Furthermore, a high-water flux was maintained until the concentration of the [N_4444_][TMBS]-based DS reached 40 wt%. The lean DS phase with a high H_2_O concentration and low viscosity covered the surface of the RO membrane during the RO treatment. Based on the results obtained in this research, the advantages of the high-temperature RO membrane treatment of the [N_4444_][TMBS]-based lean DS phase in the liquid–liquid phase separation concentration range at a temperature higher than the LCST was confirmed.

## Supplementary Materials

The following are available online at https://www.mdpi.com/article/10.3390/membranes11080588/s1, Figure S1: Determination of IL concentration required to draw water from seawater at 20 °C using (a) [P_4444_][DMBS]-based, (b) [N_4444_][TMBS]-based, and (c) [N_4444_][CF_3_COO]-based DSs. All osmotic pressure data correspond to 20 °C. The osmotic pressure of seawater at 20 °C was set to 30 bar. Figure S2: Determination of upper concentration limit from the relationship between osmotic pressure and IL concentration. Figure S3 Illustration of the configuration of DS in RO cell during RO treatment. (a) Case of low apparent [N4444][TMBS] concentration. In this case, DS lean phase becomes a continuous phase and contacts with the active layer of RO membrane. (b) Case of high apparent [N4444][TMBS] concentration. In this case, DS rich phase becomes a continuous phase and contacts with the active layer of RO membrane.
