# Supplementary material for: Using Reverse Osmosis Membrane at High Temperature for Water Recovery and Regeneration from Thermo-Responsive Ionic Liquid-Based Draw Solution for Efficient Forward Osmosis
Source: Membranes (Basel). 2021 Jul 31;11(8):588. doi: 10.3390/membranes11080588 (PMC8399973; doi:10.3390/membranes11080588)
Supplement: Supplementary file 1 [file membranes-11-00588-s001.zip › membranes-1308181-supplementary.pdf]

SUPPORTING INFORMATION

Using reverse osmosis membrane at high temperature for water recovery and regeneration from thermo-responsive ionic liquid-based draw solution for efficient forward osmosis

Eiji Kamio[a], Hiroki Kurisu[a], Tomoki Takahashi[a], Atsushi Matsuoka[a], Tomohisa Yoshioka[b], Keizo Nakagawa[b], Hideto Matsuyama[a]

a Research Center for Membrane and Film Technology, Department of Chemical Science & Engineering, Kobe University, 1-1 Rokkodai, Nada, Kobe 657-8501, Japan.
b Research Center for Membrane and Film Technology, Graduate School of Science, Technology, and Innovation, Kobe University, 1-1 Rokkodai, Nada, Kobe 657-8501, Japan

Table of contents

(1) Determination of IL concentration required to draw water from seawater using different IL-DSs 1
(2) Determination of upper concentration limit of IL-DS 2
(3) Speculated configuration of DS in RO cell during RO treatment 3

(1) Determination of IL concentration required to draw water from seawater using different IL-DSs

The minimum IL concentration of IL-DS required to draw water from seawater in FO process was determined from the relationship between the osmotic pressure of the IL-DS and IL concentration at 20 ºC (see the following Figure S1). In this work, the osmotic pressure of seawater was regarded as 30 bar.

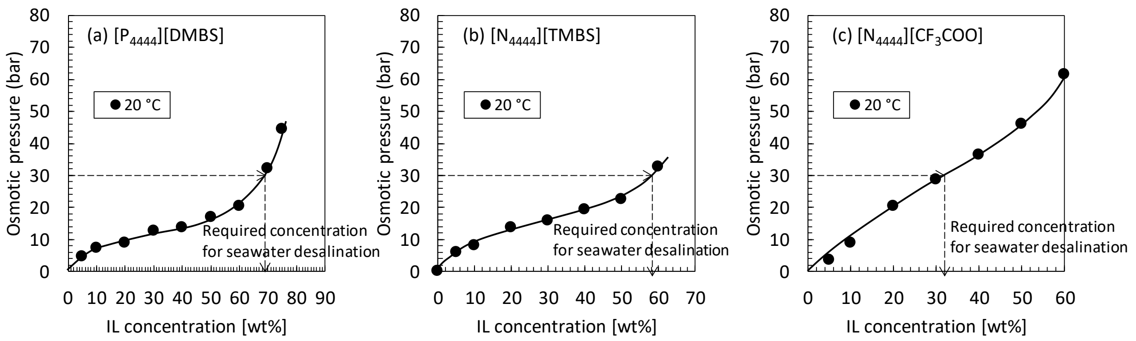

Figure S1. Determination of IL concentration required to draw water from seawater at 20 °C using (a) $[P_{4444}]$[DMBS]-based, (b) $[N_{4444}]$[TMBS]-based, and (c) $[N_{4444}][CF_3COO]$-based DSs. All osmotic pressure data correspond to 20 °C. The osmotic pressure of seawater at 20 °C was set to 30 bar.

(2) Determination of upper concentration limit of IL-DS

For different temperatures, the upper concentration limits of IL-DSs were determined from the concentration of IL of the IL-DS showing the osmotic pressure equals to the applied mechanical pressure for the RO treatment. The following figure shows an example of $[P_{4444}][DMBA]$-based DS treated at 15 bar of mechanical pressure for the RO treatment at 50 °C.

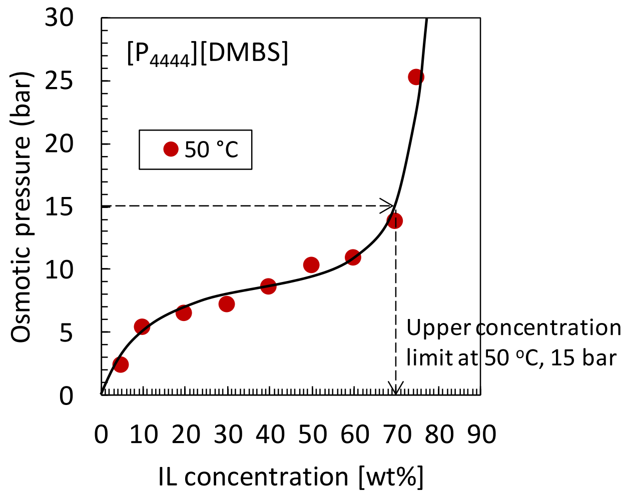

Figure S2. Determination of upper concentration limit from the relationship between osmotic pressure and IL concentration.

(3) Speculated configuration of DS in RO cell during RO treatment

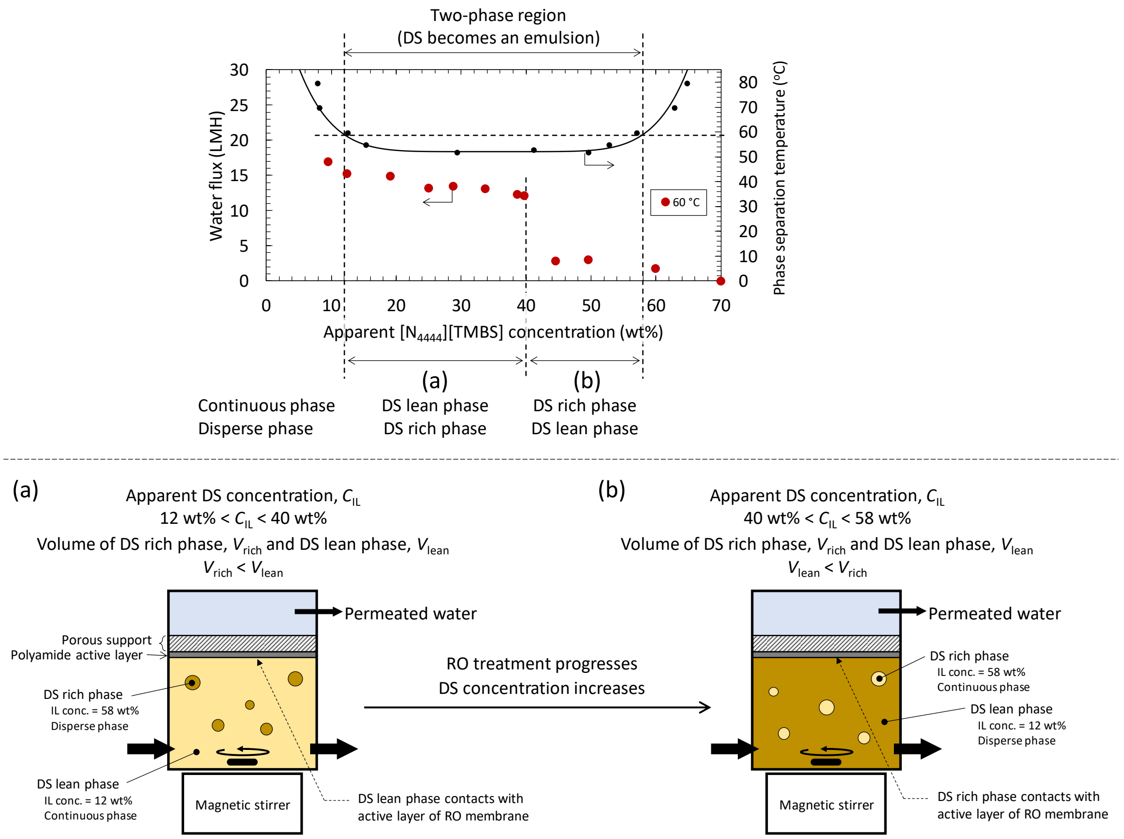

Figure S3 Illustration of the configuration of DS in RO cell during RO treatment. (a) Case of low apparent [N_{4444}][TMBS] concentration. In this case, DS lean phase becomes a continuous phase and contacts with the active layer of RO membrane. (b) Case of high apparent [N_{4444}][TMBS] concentration. In this case, DS rich phase becomes a continuous phase and contacts with the active layer of RO membrane.
